# Glutamate shall not pass: a mechanistic role for astrocytic O-GlcNAc transferase in stress and depression

**DOI:** 10.1172/JCI168662

**Published:** 2023-04-03

**Authors:** Sam E.J. Paton, Caroline Menard

**Affiliations:** Department of Psychiatry and Neuroscience, Faculty of Medicine and CERVO Brain Research Center, Université Laval, Quebec City, Quebec, Canada.

## Abstract

Major depressive disorder, characterized by aberrant glutamatergic signaling in the prefrontal cortex (PFC), is a leading cause of disability worldwide. Depression is highly comorbid with metabolic disorders, but a mechanistic link is elusive. In this issue of the *JCI*, Fan and coauthors report that elevated posttranslational modification with the glucose metabolite *N*-acetylglucosamine (GlcNAc) by O-GlcNAc transferase (OGT) contributed to stress-induced establishment of depression-like behaviors in mice. This effect was specific to medial PFC (mPFC) astrocytes, with glutamate transporter-1 (GLT-1) identified as an OGT target. Specifically, O-GlcNAcylation of GLT-1 resulted in diminished glutamate clearance from excitatory synapses. Further, astrocytic OGT knockdown restored stress-induced deficits in glutamatergic signaling, promoting resilience. These findings provide a mechanistic link between metabolism and depression and have relevance for antidepressant targets.

## Major depressive disorder and metabolic deficits

The proportion of the global population living with major depressive disorder (MDD) is estimated at 322 million people (4.4%), with an additional 260 million (3.6%) being affected by anxiety disorders (1), which are often comorbid with depression. MDD-associated symptoms and treatment responses are highly heterogenous, representing a challenge for understanding the pathogenesis of this mood disorder. While the neurobiology underlying MDD symptomatology is not completely elucidated, a major hallmark is dysregulation of monoamine (serotonin, dopamine, norepinephrine) neurotransmission in midbrain circuits responsible for emotion regulation and reward responses (2, 3).

Evidence supports that this dysfunction is due to erratic excitatory glutamatergic activity in the prefrontal cortex (PFC), a brain region involved in regulation of social behavior and emotion, resulting in a loss of top-down control of midbrain activity (4). However, antidepressant drugs targeting synapses have proven only modestly effective, with a substantial proportion of treatment resistance, suggesting underlying factors remain unaddressed (5). Interestingly, MDD has been linked to metabolic disturbances (6) and is highly comorbid with disorders characterized by disruption of glucose metabolism, including diabetes (7). Positron emission tomography scanning of individuals with depression reveals diminished glucose metabolism in the PFC (8, 9), but a direct link between deficient glucose metabolism and depressive symptoms has yet to be determined.

While glucose is mainly used in the brain for energy generation, a small amount is redirected through the hexosamine biosynthesis pathway, which produces *N*-acetylglucosamine (GlcNAc). This glucose derivative is appended onto certain proteins in a posttranslational modification referred to as O-GlcNAcylation and catalyzed by the O-GlcNAc transferase (OGT) enzyme. OGT is expressed ubiquitously, but its level is about ten times higher in the brain than in the periphery. OGT activity is essential during embryogenesis and development (10), and loss of OGT expression in adulthood leads to progressive neurodegeneration (11). Furthermore, experimental manipulation of O-GlcNAcylation can influence depressive and anxiety-like behavior in rodents (12), suggesting a role for OGT in regulating affective neural circuits. This role is supported by recent findings highlighting alterations in the brain of O-GlcNAcylation profiles in a rat model of depression (13). Nevertheless, a mechanistic pathway linking OGT to MDD pathogenesis has remained to be described.

## Astrocytic glutamate reuptake and MDD

Astrocytes are crucial mediators of cerebral homeostasis, performing diverse functions ranging from transfer of nutrients between blood vessels and neurons to regulation of neurotransmission (14). Each astrocyte can contact up to 100 synapses, with fine processes ensheathing both pre- and postsynaptic boutons to monitor and manipulate neurotransmitter concentrations in the synaptic cleft (15). Recent evidence points to astrocyte dysfunction as a mechanism underlying the disrupted neuronal circuitry associated with depression. First, postmortem tissues from individuals with MDD display reduced astrocyte densities throughout the brain (14, 16) and diminished astrocyte-blood vessel contacts (14, 17). In rodents, chronic stress exposure, which is the main environmental risk factor for developing MDD, induces more transcriptional changes in astrocytes than other cell types, particularly in the medial PFC (mPFC) (18). Exposure to chronic stress also disturbs connections between astrocytes and neurons, impairing metabolite shuttling through the brain (19). Interestingly, astrocytic dysfunction in MDD has been shown to occur in tandem with alterations in glutamate-related signaling (20), suggesting a disruption in astrocyte monitoring of glutamatergic synapses.

Glutamate transporter-1 (GLT-1, also known as EAAT2) is a sodium-dependent glutamate reuptake transporter present mostly in astrocyte plasma membranes and, to a lesser extent, in neurons (21). In astrocytes, this transporter is localized at endfeet processes ensheathing synapses, where it assists in clearing glutamate from the synaptic cleft to maintain low, nontoxic levels of this excitatory neurotransmitter (22). Further, astrocytes convert this glutamate into nonactive glutamine, which can be cycled back to neurons for reconversion to glutamate, reducing the metabolic demand of constant firing. Thus, via GLT-1, astrocytes actively contribute to maintaining optimal conditions for synaptic neurotransmission and plasticity, exerting a substantial influence on cognition and behavior (22). Indeed, manipulation of glutamate uptake through GLT-1 can influence affective behavior in rodents. For example, pharmacological inhibition of GLT-1 in the PFC induces anhedonia in rats (23), a hallmark symptom of MDD defined as the inability to feel pleasure, while GLT-1 knockdown in the same brain region in mice promotes glutamate dysfunction and depressive symptoms (4). GLT-1 is also reduced in postmortem brain tissue from individuals diagnosed with depression (20), an observation that corresponds with reports of increased extracellular glutamate in the mPFC in MDD, as measured by magnetic resonance spectroscopy (24).

## O-GlcNAcylation of GLT-1 regulates depressive behaviors in mice

**Figure 1:**
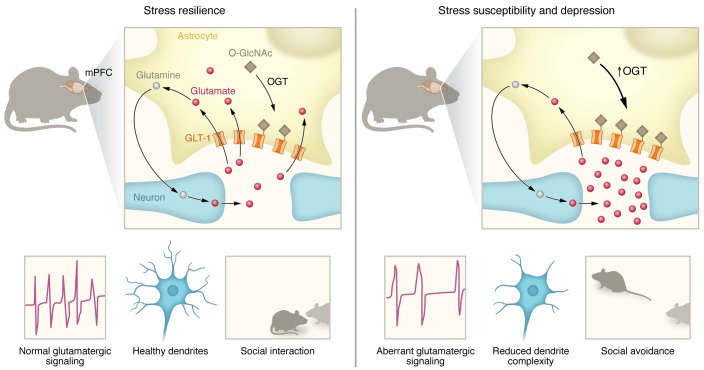
Increased OGT in mPFC astrocytes mediates stress susceptibility and depression. In healthy individuals and stress-resilient mice, astrocytes remove glutamate from synapses via GLT-1 to regulate glutamatergic neurotransmission. Importantly, this glutamate is converted to glutamine and shuttled back to neurons for reconversion to glutamate, thereby ensuring a continual supply of this essential neurotransmitter. Fan et al. (25) show that in men with depression and stress-susceptible male mice, OGT levels are increased in the PFC, a brain area involved in executive functions, social interactions, and mood regulation. This change is specific to astrocytes where the OGT enzyme catalyzes O-GlcNAcylation of GLT-1, modulating glutamate transport at the tripartite synapse. An elevated rate of this OGT-mediated posttranslational modification inhibits GLT-1 activity, leading to an increase in glutamate level in the synaptic cleft in the short-term, while simultaneously limiting transfer of glutamine to neurons and possibly reducing long-term glutamate availability. The resulting aberrant glutamatergic signaling causes reductions in dendrite complexity and depression-like symptoms, including social avoidance.

In this issue of the *JCI*, Fan et al. (25) describe a mechanistic link between altered OGT-mediated O-GlcNAcylation and abnormal glutamate neurotransmission in a mouse model of depression (Figure 1). Initially, the authors discovered that OGT mRNA was upregulated in the blood as well as in the mPFC of men with MDD. To investigate the underlying biological mechanisms and possible contribution to this mood disorder pathogenesis, they took advantage of the chronic social-defeat stress (CSDS) model, a well-established protocol inducing depression- and anxiety-like behaviors by subjecting mice to repeated physical altercation with a larger, aggressive animal (26). After 10 days of CSDS sessions, a social-interaction test is performed that allows for discrimination of two subsets of stressed animals: stress susceptible, which display social avoidance along with other maladaptive behaviors associated with MDD; and resilient, which exhibit behavioral features comparable to those of unstressed controls. Intriguingly, Fan et al. noted that OGT mRNA and protein were upregulated in the mPFC strictly in stress-susceptible mice and that this change correlated strongly with the degree of susceptibility to CSDS. It was found that cultured astrocytes, but not neurons, upregulated OGT in response to damage. The authors followed with a clever combination of in vivo functional and pharmacological experiments using transgenic mice, viral vectors, and OGT inhibitors to bidirectionally manipulate expression and activity of OGT in astrocytes and neurons, demonstrating a causal relationship between increased astrocytic OGT and the development of depressive behaviors (25).

Based on these findings, Fan et al. (25) generated astrocytic OGT conditional knockout transgenic mice to further explore the contribution of OGT to stress resilience. With this approach, the glutamate transporter GLT-1 was identified as a target of OGT, with elevated O-GlcNAcylation observed after stress exposure only in wild-type mice. Next, the authors showed in vitro that O-GlcNAcylation of GLT-1 decreased glutamate-reuptake rate, thereby potentially increasing synaptic glutamate concentrations, firing rate, and the risk of neurotoxicity. To validate this hypothesis in vivo*,* an impressive array of morphological, functional, and biochemical assays was conducted, which confirmed that OGT knockout in astrocytes protected neurons in the mPFC from stress-related damage and prevented aberrant glutamatergic signaling as well as loss of dendritic complexity and spine density.

## Clinical implications for human MDD

The Fan et al. study (25) elegantly bridges two distinct concepts of neurobiological research on depression, namely dysregulation of OGT and O-GlcNAcylation in the brain (11–13) and GLT-1 inhibition resulting in loss of astrocytic control over synapses (4, 20, 23), deepening our understanding of the cellular mechanisms underlying stress-related cognitive deficits and possibly MDD. It establishes a molecular pathway connecting stress-induced metabolic disruption, elevated O-GlcNAcylation, and loss of astrocytic control over excitatory glutamate transmission leading to behavioral deficits. The immediate clinical implication relates to O-GlcNAcylation of GLT-1 in the mPFC as an alternative antidepressant target, since monoamine-associated antidepressant drugs are helpful for many, but unfortunately not all, cases of MDD (5). However, additional research is required to fully decipher the role of OGT-mediated O-GlcNAcylation and GLT-1 in health and disease. It must be noted that OGT inhibitors may not represent a viable therapeutic avenue, since long-term loss of O-GlcNAcylation (beginning after 6 weeks) in the frontal cortex leads to neurodegeneration and memory deficits in mice (11). Moreover, in a nonstressful environment, antidepressant effects have been reported following increased O-GlcNAcylation (12), suggesting that this system could play an important role in mood regulation under physiological conditions. On the other hand, GLT-1 activity is heavily regulated in cortical astrocytes, with several other posttranslational modifications involved (21). Over longer time periods, astrocytes could theoretically compensate for changes in O-GlcNAcylation of GLT-1 by adapting the rate of other modifications, potentially complicating efficient drug development. Traditionally, MDD treatment is necessary for several months, with a high prevalence of relapse, and thus, it will be important in the future to explore whether targeting OGT-mediated O-GlcNAcylation could have beneficial long-lasting effects.

## Conclusion and future directions

The report of Fan et al. (25) links what were previously considered distinct systems to gain mechanistic insights and shed light on glial regulation of stress-related neurobiology and MDD. However, many unknowns still remain. It will be important to investigate and identify the molecular and cellular mechanisms underlying selective upregulation of OGT expression in astrocytes of the mPFC following stress and determine whether this phenomenon is limited to this brain area or certain types or durations of stress. Moreover, this research was conducted mainly in males. Though sex-specific symptomatology, prevalence, and treatment responses are observed with MDD, the findings cannot necessarily be translated across sexes. As OGT is an X-linked gene (10), its potential effects in women and female mice, if any, should be considered in future studies.

## Version 1 04/03/2023

Electronic publication

## References

[1] Friedrich MJ (2017). Depression is the leading cause of disability around the world. JAMA.

[2] Mayberg HS (1997). Limbic-cortical dysregulation: a proposed model of depression. J Neuropsychiatry Clin Neurosci.

[3] Russo SJ, Nestler EJ (2013). The brain reward circuitry in mood disorders. Nat Rev Neurosci.

[4] Fullana MN (2019). In vivo knockdown of astroglial glutamate transporters GLT-1 and GLAST increases excitatory neurotransmission in mouse infralimbic cortex: Relevance for depressive-like phenotypes. Eur Neuropsychopharmacol.

[5] Leichsenring F (2022). The efficacy of psychotherapies and pharmacotherapies for mental disorders in adults: an umbrella review and meta-analytic evaluation of recent meta-analyses. World Psychiatry.

[6] Chan KL (2019). Central and peripheral inflammation link metabolic syndrome and major depressive disorder. Physiology (Bethesda).

[7] Anderson RJ (2001). The prevalence of comorbid depression in adults with diabetes: a meta-analysis. Diabetes Care.

[8] Baxter LR (1989). , et al. Reduction of prefrontal cortex glucose metabolism common to three types of depression. Arch Gen Psychiatry.

[9] Kennedy SH (2001). Changes in regional brain glucose metabolism measured with positron emission tomography after paroxetine treatment of major depression. Am J Psychiatry.

[10] Shafi R (2000). The O-GlcNAc transferase gene resides on the X chromosome and is essential for embryonic stem cell viability and mouse ontogeny. Proc Natl Acad Sci U S A.

[11] Wang AC (2016). Loss of O-GlcNAc glycosylation in forebrain excitatory neurons induces neurodegeneration. Proc Natl Acad Sci U S A.

[12] Cho Y (2020). Elevated O-GlcNAcylation induces an antidepressant-like phenotype and decreased inhibitory transmission in medial prefrontal cortex. Sci Rep.

[13] Liu W (2018). OGT-related mitochondrial motility is associated with sex differences and exercise effects in depression induced by prenatal exposure to glucocorticoids. J Affect Disord.

[14] Dion-Albert L (2023). Neurovascular adaptations modulating cognition, mood, and stress responses. Trends Neurosci.

[15] Lyon KA, Allen NJ (2021). From synapses to circuits, astrocytes regulate behavior. Front Neural Circuits.

[16] O’Leary LA, Mechawar N (2021). Implication of cerebral astrocytes in major depression: A review of fine neuroanatomical evidence in humans. Glia.

[17] Rajkowska G (2013). Coverage of blood vessels by astrocytic endfeet is reduced in major depressive disorder. Biol Psychiatry.

[18] Labonte B (2017). Sex-specific transcriptional signatures in human depression. Nat Med.

[19] Murphy-Royal C (2020). Stress gates an astrocytic energy reservoir to impair synaptic plasticity. Nat Commun.

[20] Choudary PV (2005). Altered cortical glutamatergic and GABAergic signal transmission with glial involvement in depression. Proc Natl Acad Sci U S A.

[21] Peterson AR, Binder DK (2019). Post-translational regulation of GLT-1 in neurological diseases and its potential as an effective therapeutic target. Front Mol Neurosci.

[22] Murphy-Royal C (2015). Surface diffusion of astrocytic glutamate transporters shapes synaptic transmission. Nat Neurosci.

[23] John CS (2012). , Cohen BM, et al. Blockade of astrocytic glutamate uptake in the prefrontal cortex induces anhedonia. Neuropsychopharmacology.

[24] Kantrowitz JT (2021). Ventromedial prefrontal cortex/anterior cingulate cortex Glx, glutamate, and GABA levels in medication-free major depressive disorder. Transl Psychiatry.

[25] Fan J (2023). GlcNAc transferase in astrocytes modulates stress susceptibility through glutamatergic synaptic transmission. J Clin Invest.

[26] Golden SA (2011). A standardized protocol for repeated social defeat stress in mice. Nat Protoc.

